# Extensive Aplasia Cutis Congenita Encircling the Trunk Associated with Fetus Papyraceus

**DOI:** 10.1155/2020/8824757

**Published:** 2020-08-27

**Authors:** Alexander K. C. Leung, Kin Fon Leong, Joseph M. Lam

**Affiliations:** ^1^The University of Calgary, Calgary, AB T2N 1N4, Canada; ^2^The Alberta Children's Hospital, Calgary, Alberta, T2M 0H5, Canada; ^3^Pediatric Institute, Kuala Lumpur General Hospital, Kuala Lumpur, Malaysia; ^4^Department of Dermatology and Skin Sciences, University of British Columbia, Vancouver, V6T 1Z4, Canada; ^5^BC Children's Hospital Vancouver, Vancouver, BC V6H 3V4, Canada

## Abstract

Aplasia cutis congenita associated with fetus papyraceus, though rare, is well known. On the other hand, aplasia cutis congenita associated with fetus papyraceus presenting with symmetrical circumferential scarring encircling the trunk has not been previously reported. Herein, we report a 2-month-old girl with symmetrical circumferential scarring encircling the trunk associated with fetus papyraceus.

## 1. Introduction

Aplasia cutis congenita associated with fetus papyraceus (vanishing twin or mummified dead fetus), though rare, is a well-known phenomenon. To our knowledge, aplasia cutis congenita with symmetrical circumferential scarring encircling the trunk associated with fetus papyraceus has not been previously reported. Herein, we report a 2-month-old girl with extensive aplasia cutis congenita encircling the trunk. She was a survivor of a twin pregnancy; the co-twin died at a gestational age of 11 weeks.

## 2. Case Report

A 2-month-old girl presented to us with a history of a skin defect encircling her trunk since birth. The 28-year-old primigravida mother was healthy with no history exposure to drugs, infectious diseases, or trauma during pregnancy. Antenatal ultrasonography at 11 weeks of gestational age showed demise of one of the two monochorionic fetuses. The rest of the pregnancy was uncomplicated. The infant was delivered at 36 weeks of gestation following a normal spontaneous vaginal delivery. The Apgar scores were 6 and 9 at 1 minute and 5 minutes, respectively. Her birth weight was 2.5 kg, length 49.5 cm, and head circumference 34.7 cm. Parents denied any history of consanguinity, similar skin conditions, or blistering disorders.

**Figure 1:**
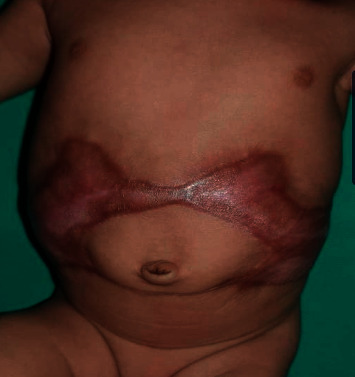
Aplasia cutis congenita presenting as a bilateral symmetrical well-demarcated butterfly-shaped (or stellate) pattern on the abdomen extending to the back.

**Figure 2:**
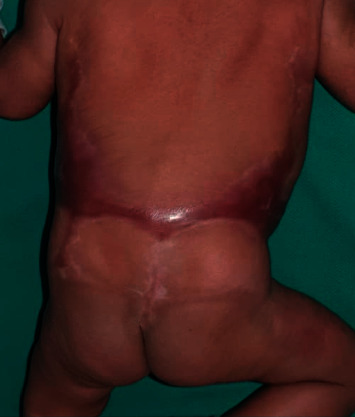
Aplasia cutis congenita in an H-shaped distribution on the back.

Examination revealed well-demarcated, symmetrical, circumferential scarring encircling the trunk (Figures 1 and 2). The vital signs (temperature 36.8°C, heart rate 115 beats per minute, and respiratory rate 35 breaths per minute) were normal. The rest of the examination was unremarkable. In particular, there was no evidence of other congenital abnormalities.

Given the history of spontaneous intrauterine demise of the co-twin at 11 weeks' gestational age and the finding of symmetrical truncal aplasia cutis congenita, a diagnosis of aplasia cutis congenita associated with fetus papyraceus was made. The patient was referred to a plastic surgeon for scar revision. The parents were happy with the esthetic outcome. There was no functional impairment.

## 3. Discussion

Aplasia cutis congenita is a heterogeneous group of rare disorders characterized by a localized or widespread, complete or partial absence of different layers of the skin at birth, occasionally extending to the bone [1, 2]. This phenomenon was first described by Cordon in 1767 [3]. The condition can present at birth with scarring which represents lesions that have already healed *in utero* or with glistening absence of the skin which manifests as well-demarcated, translucent, ulcerated membranes through which the underlying structures can be visualized [4]. In the latter case, the lesions will eventually heal with scarring. The diagnosis of aplasia cutis congenita is mainly clinical. In 1986, Ilona Frieden classified aplasia cutis congenita into 9 groups, depending on the site of the skin defect, associated anomalies, associated syndromes, underlying causes, and teratogens as causative agents as follows [1]:

- Group 1: aplasia cutis congenita of the scalp without multiple anomalies
- Group 2: aplasia cutis congenita of the scalp associated with limb anomalies
- Group 3: aplasia cutis congenita of the scalp associated with epidermal and organoid nevi
- Group 4: aplasia cutis congenita overlying embryologic malformations
- Group 5: aplasia cutis congenita associated with fetus papyraceus or placental infarcts
- Group 6: aplasia cutis congenita associated with epidermolysis bullosa
- Group 7: aplasia cutis congenita of the extremities without blistering
- Group 8: aplasia cutis congenita caused by specific teratogens
- Group 9: aplasia cutis congenita associated with malformation syndromes

Our patient had aplasia cutis congenita associated with fetus papyraceus and fits into group 5 of Frieden's classification. While 70 to 85% of individuals with aplasia cutis congenita have lesions localized to the vertex of the scalp, truncal aplasia cutis congenita, especially linear, bilateral, and symmetrical lesions with a stellate pattern, butterfly pattern, or “H” configuration, is typically associated with fetus papyraceus [5–8]. Truncal aplasia cutis congenita associated with fetus papyraceus is most common in monochorionic pregnancies, with loss of a sibling fetus at around the 12^th^ to 14^th^ week of gestation [6, 9]. While death of the co-twin in the late first or early second trimester leads to complete resorption of fetus papyraceus (vanishing twin) as is in the present case, later death results in mummification of the dead fetus [7].

In 2015, Meena et al. reported a newborn infant, a survivor of twin pregnancy, who had aplasia cutis congenita with symmetrical, stellate pattern involvement on the back, radiating laterally to both arms till elbow joints, both thighs till knee joints, and anteriorly to involve the trunk but sparing the subcostal angle [7]. Our case is unique in that the aplasia cutis congenita presented with symmetrical circumferential scarring encircling both the back and anterior trunk. To our knowledge, this has not been previously reported.

Presumably, aplasia cutis congenita results from disrupted development or degeneration of the skin *in utero*, leading to scarring or absence of the skin at birth [4, 10, 11]. The vascular theory suggests that the death of one twin *in utero* allows passage of thrombogenic materials to the living twin through placental vascular anastomoses, activating the coagulation cascade in the living twin and resulting in disseminated intravascular coagulation with ischemia and infraction of the developing skin [5–7]. This theory is not supported by the fact that fetal blood sampling immediately before and within 24 hours of death in monochorionic twin pregnancies complicated by single intrauterine death did not reveal abnormal coagulation profiles [12, 13]. Intrauterine trauma is also a possibility although majority of cases do not have a history of intrauterine trauma. More likely, decreased or falling blood pressure of the dying twin leads to exsanguination and acute hypovolemia of the surviving twin with resultant ischemia of the skin and consequent aplasia cutis congenita [4, 10, 13, 14]. The characteristic involvement of the trunk and extremities is thought to represent watershed areas that are farthest from the vascular supply and therefore most susceptible to ischemic insults in the setting of hypovolemia [8, 15]. In this regard, Doppler ultrasound has demonstrated acute transfusion from the surviving to the dying twin [16].

## 4. Conclusion

We report a 2-month-old girl with an extensive aplasia cutis congenita encircling the trunk. She was a survivor of a twin pregnancy; the co-twin died at a gestational age of 11 weeks. To our knowledge, aplasia cutis congenita associated with fetus papyraceus presenting with symmetrical circumferential scarring encircling the trunk has not been previously reported.
